# Electrically Conductive Nanoparticle-Enhanced Epoxy Adhesives for Localised Joule Heating-Based Curing in Composite Bonding

**DOI:** 10.3390/polym17091176

**Published:** 2025-04-25

**Authors:** Karina Dragasiute, Gediminas Monastyreckis, Daiva Zeleniakiene

**Affiliations:** Department of Mechanical Engineering, Kaunas University of Technology, Studentu str. 56, LT-51424 Kaunas, Lithuania; karina.dragasiute@ktu.edu (K.D.); daiva.zeleniakiene@ktu.lt (D.Z.)

**Keywords:** adhesives, curing, carbon nanotube, thermal imaging analysis, finite element analysis, shear strength

## Abstract

This study investigates the application of carbon nanotube (CNT)-enhanced epoxy adhesives for localised Joule heating-based curing in composite bonding. The electrical, thermal, and mechanical properties of epoxy with 0.25–1 wt% CNT loadings were evaluated. A simple CNT alignment method using DC voltage showed improved electrical conductivity, greatly reducing the percolation threshold. Transient thermal analysis using finite element modelling of representative volume elements revealed that aligned CNTs led to increased localised temperatures near the CNT clusters. The model was validated with infrared thermal imaging analysis, which also showed similar non-linear heat distribution and more uniform heating under higher CNT loading. Additionally, power distribution mapping was evaluated through inverse modelling techniques, suggesting different conductivity zones and cluster distribution within the single-lap joint. The numerical and experimental results demonstrated that CNT alignment significantly enhanced localised conductivity, thereby improving curing efficiency at lower voltages. The lap shear test results showed a peak shear strength of 10.16 MPa at 0.5 wt% CNT loading, 9% higher than pure epoxy. Scanning electron microscopy analysis confirmed the formation of aligned CNT clusters, and how CNT loading affected the failure modes, transitioning from cohesive to void-rich fracture patterns at a higher wt%. These findings establish CNT-enhanced Joule heating as a viable and scalable alternative for efficient composite bonding in aerospace and structural applications.

## 1. Introduction

According to life cycle assessments of structural aircraft materials, the usage of composite materials significantly lowers the weight of an aircraft, which in turn decreases fuel consumption and therefore reduces emissions [1,2]. Despite these advantages, one of the primary challenges facing composite materials in aerospace is the bonding process, which is critical for ensuring structural integrity. Traditional methods rely on two-part epoxy adhesives that require curing at high temperatures, often in large ovens explicitly designed for this purpose [3,4]. Conventional curing techniques are energy-intensive, costly, and environmentally unsustainable [3,5]. Methods such as oven and autoclave curing necessitate very slow heat-up rates (1–2 °C/min) due to the low thermal conductivity of polymers [6,7]. This results in long curing cycles and high energy consumption, especially for thick composite laminates where non-homogeneous curing and significant thermal stresses are observed [6,8,9].

Additionally, beyond energy concerns, the scalability of traditional curing methods is further limited by their inability to effectively handle large-scale composite components, thereby restricting modern aviation manufacturing [4,6]. The need to use large curing ovens adds complexity when manufacturing oversized parts, as the process cannot effectively accommodate the growing size of composite components [3,6,10]. As Mahdi et al. noted, oven curing struggles with thick composite layers, leading to inefficient heat diffusion [3]. Energy-efficient alternatives like resistance heating are essential for reducing costs and improving scalability.

Given these limitations, alternative approaches must address energy consumption, scalability, and the preservation of material properties. The bonding method using localised Joule’s heating improves efficiency by reducing curing time and energy usage through resistance heating, with energy savings varying by method and material. The proposed method speeds up the polymerisation process of the adhesives by using targeted heat energy. It avoids excessive heating of the surrounding materials, thereby preserving their mechanical properties. The method involves embedding carbon nanotubes (CNTs) into the adhesive layer, which generates heat directly when an electrical current is applied. CNTs are widely used nanoparticles that have shown promising results in large-scale applications of electrically conductive multifunctional composites [11,12].

CNT-based resistive heating demonstrates comparable fracture toughness to oven-cured adhesives, with energy usage optimised for localised areas. CNTs are highly conductive, both thermally and electrically, allowing us to target the bonding area and limit unnecessary heating of the surrounding materials, which helps preserve the composite properties [3,13,14,15]. Uniform CNT distribution is critical for maintaining the structural integrity of bonded components, as inconsistent heating can lead to weak spots that could compromise the strength of the structure [8,16]. CNTs and other nanoparticles offer significant advantages for improving mechanical performance, including increased strength and fatigue life [1,2,10,14,17,18]. CNT-enhanced adhesives showed improved bond strength by up to 10%, providing enhanced durability under high stress and in harsh environments compared to conventional epoxy adhesives [19,20]. In addition, a study showed that adding 0.5 wt% single-walled CNT to the epoxy adhesive improves the peel strength by 30% compared to the baseline adhesive. However, for 1 wt% reinforcement, while peel strength increased by 30%, the lap shear strength was reduced by 10–15% [21]. This suggests that while CNTs benefit the bonding performance considerably, excessive loading can negatively impact specific mechanical properties.

Although CNT-enhanced adhesives provide significant mechanical and thermal benefits, controlling CNT orientation—whether randomly dispersed or aligned—further influences conductivity, stress distribution, and overall adhesive performance. Aligned CNTs create continuous conductive pathways, resulting in enhanced electrical conductivity compared to randomly oriented CNTs. For instance, aligned CNT composites exhibit up to 360% higher conductivity along the alignment direction than randomly dispersed CNT networks [22]. This improvement is attributed to the reduced contact resistance and increased charge carrier mobility. Additionally, electron transport in CNT networks is highly dependent on junction resistance, with aligned CNTs minimising the number of tube-to-tube junctions, thereby lowering overall electrical resistance [23]. Mechanically, CNT alignment facilitates more effective load transfer, increasing up to 49% in modulus compared to composites with randomly oriented CNTs [24]. Furthermore, damping properties are significantly enhanced with CNT alignment, with studies demonstrating up to 37% improvement in structural damping in aligned CNT-reinforced composites [25]. This improvement is critical for applications requiring vibration damping and dynamic stability, such as aerospace and automotive structures.

Electrical conductivity in CNT composites is influenced by alignment and the interplay between electron tunnelling and conductive network formation. The percolation threshold determines the dominant electrical transport mechanism [26]. At low CNT concentrations, electron tunnelling governs conductivity, whereas, at higher concentrations, percolation leads to the formation of conductive networks, significantly enhancing electrical performance. This percolation behaviour is also highly sensitive to CNT aspect ratio, as longer CNTs form percolative paths at lower concentrations, thereby improving conductivity [26]. However, thermal transport in these systems remains highly anisotropic, with vertically aligned CNTs exhibiting superior heat dissipation compared to randomly oriented networks [27]. Studies indicate that partially aligned CNTs can enhance thermal conductivity up to eight times compared to isotropic CNT dispersions [24,28].

In response to these challenges, this research proposes an innovative bonding technology utilising in situ CNT alignment and localised Joule heating, enabling efficient epoxy curing. A key aspect of this study is the extensive computational modelling, which was employed to analyse thermal distribution at the nanoscale, optimise CNT loading in epoxy adhesives, and predict the thermal behaviour of the bonded joints. Finite element analysis (FEA) was performed to simulate transient heat transfer, allowing for a deeper understanding of localised thermal gradients within the CNT-enhanced adhesive layer. These numerical models were validated through infrared thermal imaging, ensuring a high correlation between the computational predictions and the experimental results.

This research differs from the previous studies by focusing on the combined effect of CNT alignment and localised heating in the adhesive bond area, rather than treating them separately. Unlike traditional CNT-reinforced adhesives, which often suffer from high percolation thresholds and inconsistent conductivity, this study demonstrates that strategic CNT alignment significantly enhances localised heating efficiency, leading to faster and more energy-efficient curing cycles. Additionally, while most prior work has focused on bulk CNT-epoxy composites, this study provides a detailed analysis of the nanoscale conductive network within an adhesive joint, highlighting how CNT distribution affects both curing dynamics and mechanical performance. The findings establish CNT-enhanced Joule heating as a scalable alternative to conventional oven-based curing.

## 2. Materials and Methods

### 2.1. Materials

A two-component epoxy system was used as the polymer matrix, consisting of CHS Epoxy 582 resin, which was modified with a reactive diluent (1,4-butanediol diglycidyl ether) for optimal viscosity. The hardener, Telalit 0420 (isophorone diamine), was added in a 100:25 weight ratio. All components were supplied by Spolchemie (Ústí nad Labem, Czech Republic).

NC7000 multi-walled CNTs (Nanocyl, Sambreville, Belgium) were used in powder form, with an average diameter of 9.5 nm and size of 1.5 µm.

Glass fibre-reinforced plastic (GFRP) composites were fabricated using 12 layers of 163 g/m^2^ plain weave Interglas 92105, aero, FK 144 glass fabric (R&G Faserverbundwerkstoffe GmbH, Waldenbuch, Germany). The matrix was Biresin CR-122-5 epoxy (Sika AG, Zürich, Switzerland) combined with an amine curing agent in a 10:3 weight ratio. The curing process included an overnight cure at room temperature, followed by four hours of post-curing at 100 °C in a convection oven.

For the technical implementation of electrical conductivity, 8 g/m^2^ non-woven carbon fibre chopped strand mat (CSM) (R&G Faserverbundwerkstoffe GmbH, Waldenbuch, Germany) was applied as the final layer of the specimens. For the same reason, silver conductive ink (Thermo Scientific, Karlsruhe, Germany) was applied at electrode connection points.

### 2.2. Epoxy/CNT Masterbatch Preparation

To prepare the epoxy/CNT masterbatch, a specific procedure was used [11,29]. First, 1.3 g of CNTs were added to 1000 mL of hexane (Sigma-Aldrich, Darmstadt, Germany) and mixed via sonication (UP400S, Hielscher Ultrasonics GmbH, Teltow, Germany) for 15 min to achieve a well-dispersed mixture. Following this, 65 g of epoxy resin was added to the solution and manually stirred until the solution became fully transparent, indicating CNT embedment in the resin. The resulting epoxy/CNT sediment was then collected and degassed under vacuum at 50 °C for 1 h to remove any residual hexane. This process resulted in an epoxy masterbatch containing 2 wt% CNTs. For the experimental study, the epoxy/CNT masterbatch was diluted further with more epoxy resin and hardener to prepare samples with final CNT loadings of 0.25, 0.50, 0.75, and 1 wt%.

### 2.3. Specimen Preparation

**Figure 1:**
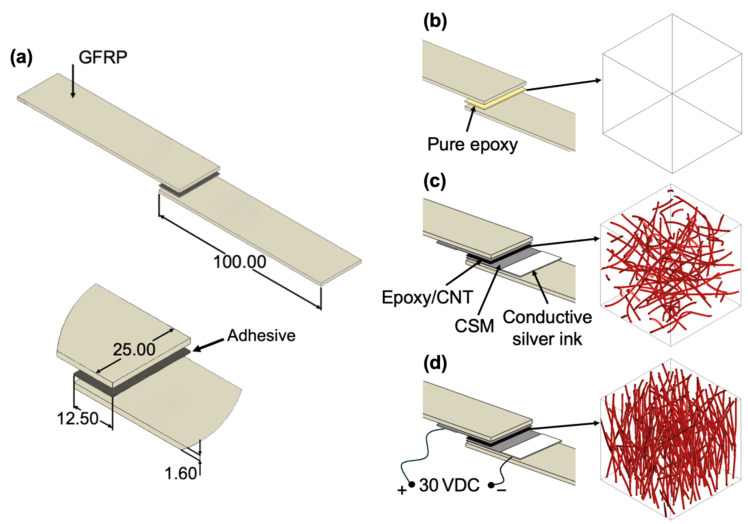
Single-lap specimen: (**a**) geometry, (**b**) pure epoxy adhesive, (**c**) randomly dispersed CNTs, (**d**) aligned CNTs in adhesive.

Specimens were produced following the ISO 4587 standards [30] for lap shear testing of rigid-to-rigid bonded assemblies. After curing, they were cut into single-lap joint specimens with the appropriate dimensions for testing (Figure 1a). All surfaces in the bonding area were cleaned with acetone prior to assembly to ensure optimal adhesion and minimise contaminants. Glass fibre spacers of 0.22 mm were used to maintain a uniform bond thickness. The overlap length was set to 12.5 ± 0.25 mm, applied with the adhesive, and clamped under consistent pressure for 24 h.

The reference specimens were fabricated without additives in the bonding area, using the pure epoxy adhesive (Figure 1b). These specimens underwent room-temperature curing for 24 h, followed by a staged post-curing cycle, consisting of 2 h at 50–60 °C, 1 h at 80 °C, and a final 1 h at 120 °C. Elevated temperatures were achieved in an oven.

The specimens with CNT-loaded adhesives (Figure 1c,d) were prepared following the standard GFRP preparation process, with the key difference that a layer of carbon fibre CSM was placed on top of the GFRP panels during manufacturing. The bonding area was lightly abraded to expose the CSM layer in the bonding area and improve electrical contact. Six specimens were prepared for each epoxy/CNT wt% loading. For the bonding area, an epoxy/CNT adhesive was applied to the exposed CSM layer, while silver conductive ink, at a distance of 1 cm from the bonding area, was used to ensure that consistent electrical conductivity is achieved at the wire–CSM contact.

Additionally, a CNT-reinforced adhesive underwent a CNT alignment process to investigate the effects of CNT orientation. The process of CNT alignment involved exposing non-cured (liquid epoxy) samples to 10–30 VDC (depending on CNT wt%) for 3 s and 10 repetitive times, which caused the CNTs to align in the direction of the current flow. The magnitude of the applied DC voltage was selected based on preliminary tests to optimise alignment efficiency while avoiding excessive heating of the uncured adhesive. Lower voltages (10–15 VDC) were used for higher CNT loadings (e.g., 0.75–1 wt%) due to their lower electrical resistance, whereas higher voltages (up to 30 VDC) were applied for lower CNT loadings to ensure sufficient electric field strength for alignment. Electrical resistance measurements were taken before and after the alignment using a Fluke 287 RMS multimeter (Fluke Corporation, Everett, WA, USA). The electrical conductivity (*σ*) was calculated according to the following formula:

(1) $$\sigma =\frac{L}{RA}$$

where *R* is the measured resistance, *L* is the length of the conductive path, and *A* is the cross-sectional area of the sample.

To provide a baseline for the comparison of mechanical testing, single-lap specimens with randomly dispersed CNTs were oven-cured under the same temperature profile as the pure epoxy and aligned CNT samples.

### 2.4. Localised Curing Procedure

**Figure 2:**
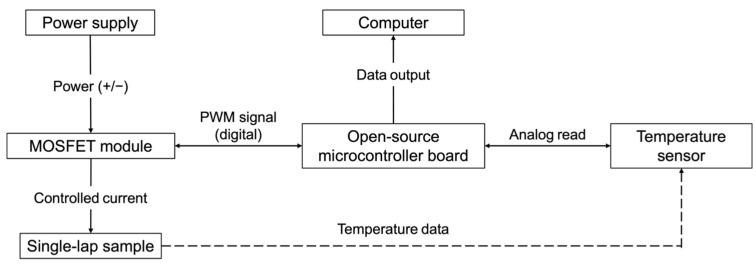
Simplified scheme of experimental setup for localised Joule heating-based curing.

Localised curing was performed using a heating system controlled by an Arduino Mega 2560 Rev3 (Arduino, Turin, Italy). Temperature sensors (LM35DZ TO92 (Texas Instruments, Dallas, TX, USA)) were attached to the top of the bonding area of each specimen to monitor and regulate the temperature throughout the staged curing cycle, ensuring precise control at each setpoint. The system was powered by a UNI-T linear laboratory power supply (0–30 VDC, 0–5 A) (UNI-T, Dongguan, China) and regulated via a semiconductor relay module with an IRF520 transistor (Iduino, Shenzhen, China), which controlled the heating elements through Pulse Width Modulation (PWM) signals. A simplified experimental setup is shown in Figure 2.

Temperature data from the sensors were processed in real time by the Arduino, which dynamically adjusted the PWM outputs to maintain the target temperature. The control code employed an analogue feedback loop, continuously fine-tuning the power input to maintain the required temperature at each stage. This approach ensured that every specimen precisely followed the pre-defined curing profile (2 h at 50–60 °C, 1 h at 80 °C, and a final 1 h at 120 °C), preventing overheating or temperature fluctuations that could affect the quality of the bonded joints.

### 2.5. Thermal Analysis

To further evaluate the curing process and ensure uniform heat distribution, a thermal imaging camera FLIR SC7000 (Teledyne FLIR LLC, Wilsonville, OR, USA), with a pixel pitch of 30 µm and a temperature accuracy of ±1%, was used to monitor thermal distribution across the bonding areas during the localised curing process (Figure S1). The camera captured real-time thermal data, providing detailed insights into localised heating effects and verifying consistent thermal application across each CNT-reinforced specimen. For the thermal analysis experiment, each specimen was heated for 15 s with 0.34 W power, ensuring precise thermal distribution.

### 2.6. Mechanical Testing

Composite samples were tested on a Tinius Olsen H10KT machine (Tinius Olsen, Horsham, PA, USA) with a load cell capacity of 10 kN, following the ISO 291 standards [31] (Figure S2). Testing was conducted at a crosshead speed of 0.5 mm/min, with the machine crosshead holding 37.5 mm on either side of each specimen. Data on load and deformation were recorded using the QMAT 5.37 software. Each specimen was tested in accordance with ISO 4587 [30] specifications to assess lap shear strength, ensuring standardised evaluation of bonding performance across all CNT loadings.

### 2.7. Post-Fracture Scanning Electron Microscopy Characterisation

The shear-strength-tested bonding area underwent scanning electron microscopy (SEM) analysis through S-3400N (Hitachi, Tokyo, Japan) with 5 kV accelerating voltage. SEM analysis of the bonded area focused on inspecting fracture surfaces to determine adhesion behaviour and void distribution within the bonded region. The captured images showed both overall failure patterns and detailed microstructural features. To further evaluate the impact of CNT content on void formation, a high-resolution Dino-Lite digital microscope (AnMo Electronics Corporation, New Taipei City, Taiwan, China) was used to capture detailed images of the epoxy and CNT-enhanced epoxy adhesives’ surfaces. The percentage of fracture surface area which consisted of voids was calculated for each CNT loading and pure epoxy specimen. The calculation area was 3 × 4 mm, and the total tested area for each specimen was 36 mm^2^. Pure epoxy was tested to provide a baseline reference.

### 2.8. Finite Element Analysis

#### 2.8.1. Model Development and Material Properties

Finite element analysis was conducted to evaluate the transient thermal response of CNT-enhanced epoxy adhesive under localised heating conditions. The simulations were performed in ANSYS Workbench 2023 R2, while the representative volume element (RVE) was generated using Hexagon Digimat FE. To analyse localised heat transfer at the nanoscale without unnecessary computational complexity, an RVE of 1000 nm^3^ was used.

Digimat FE was used to generate CNT inclusions. The final RVE for numerical calculations included only the epoxy matrix with predefined hollow regions corresponding to the CNT locations. This approach maintained the thermal influence of the CNTs without directly simulating their nanostructure, optimising computational efficiency while ensuring realistic heat transfer behaviour. The CNTs were modelled in Digimat with a diameter of 9.5 nm and a length of 1.5 μm, consistent with manufacturer specifications.

Eight models with randomly and aligned CNT distribution were developed, representing 0.25–1 wt% CNT loadings. The CNT alignment was concentrated in the central region due to the electrical field gradient and boundary constraints imposed by the electrode configuration. In the experimental setups, electric field-induced alignment was strongest in areas with higher field intensity, typically near the centre of the conductive path, while edge effects and field distortions reduced alignment uniformity near the periphery [32,33,34]. This effect was replicated in the simulation by selectively applying the alignment condition in the high-field region, ensuring consistency with experimental observations. Additionally, the alignment process was influenced by CNT mobility in the epoxy matrix, which was higher in regions where the polymer remained in a more fluid state during curing [34,35]. These factors led to a localised aligned cluster, rather than a uniform distribution across the entire adhesive layer.

The material properties for the epoxy matrix were as follows: isotropic thermal conductivity 1.9 × 10^−11^ W/(nm °C); isotropic resistivity 1.9 × 10^19^ Ω/nm; specific heat capacity 1100 J/(kg °C); and density 2 × 10^−18^ kg/nm^3^ (slightly adjusted for mass-scaling effect). CNT regions were not explicitly assigned material properties in the simulations, as their influence was incorporated through heat flux application at predefined hollowed surfaces. The CNT/epoxy interface was not separately modelled with interfacial thermal resistance, assuming efficient heat transfer between the CNTs and the surrounding matrix.

#### 2.8.2. Mesh Generation and Analysis Settings

A tetrahedral mesh was generated to ensure precise thermal modelling while maintaining computational efficiency. Medium smoothing and a slow transition were applied to improve element quality and ensure gradual size variations across the domain. The targeted element size was set to 5 nm, with finer meshing concentrated around the CNT-hollowed regions.

**Figure 3:**
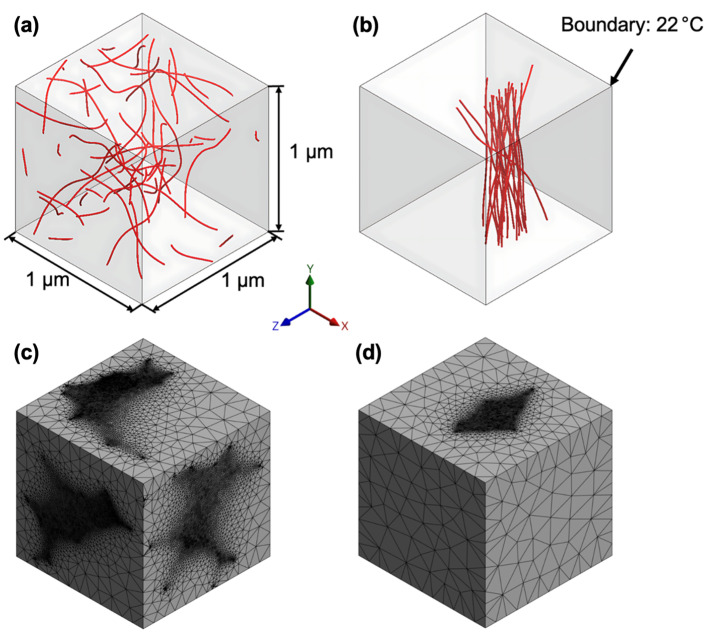
CNT distribution and meshing for 0.25 wt% CNT models: (**a**) random and (**b**) aligned CNT distributions; (**c**,**d**) corresponding meshed RVEs.

For the 0.25 wt% CNT models, the meshing resulted in 231 k elements for the random distribution and 73 k elements for the aligned configuration. For comparison, at 1 wt% CNT content, the random distribution had 511 k elements, while the aligned configuration had 323 k elements. The simulation lasted 15 s in total, with a minimum time step of 0.01 s. The model example and mesh configurations for the random and aligned 0.25 wt% CNT distributions are presented in Figure 3.

#### 2.8.3. Heat Transfer Model and Boundary Conditions

The initial temperature of the system was set at 22 °C, and a temperature boundary condition of 22 °C (Figure 3b) was imposed at the upper right corner to enable thermal calculations. A heat flux was applied for 15 s, consistent with the duration of thermal imaging experiments, where a total power of 0.34 W was applied over the adhesive overlap region. The corresponding heat flux values were calculated per RVE volume to ensure that the local thermal behaviour could be analysed under equivalent multiscale conditions.

The assigned heat flux values were 4.0 × 10^−13^ W/nm^2^ for 0.25 wt% CNT models, 2.0 × 10^−13^ W/nm^2^ for 0.5 wt%, 1.333 × 10^−13^ W/nm^2^ for 0.75 wt%, and 1.0 × 10^−13^ W/nm^2^ for 1 wt%, applied directly at the CNT hollow surfaces. This approach allowed for an investigation into how CNT distribution influenced the formation of thermal gradients and the resulting temperature distribution across the adhesive.

Since the RVE represented a periodic section of the adhesive layer, convection and radiation effects were not applied, as the model assumed that analogical repeating units surrounded the analysed volume, ensuring continuity in heat dissipation.

## 3. Results and Discussion

### 3.1. CNT Alignment and Resistance Testing

**Figure 4:**
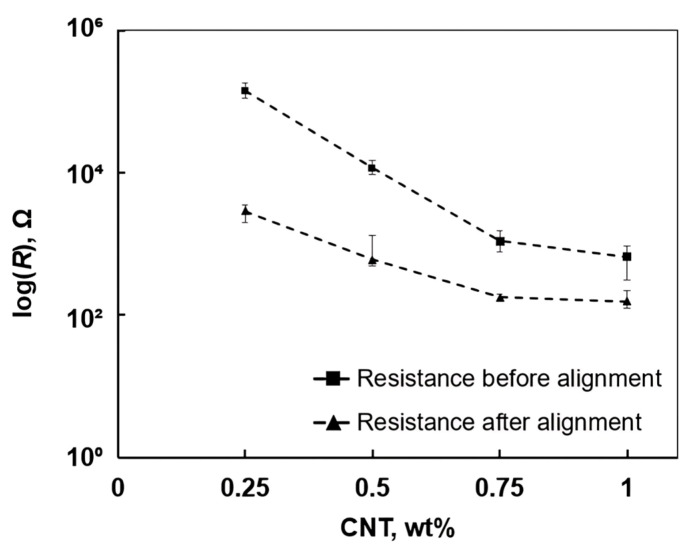
Resistance measurements before and after CNT alignment.

The average resistance before and after alignment, as summarised in Figure 4, indicates a substantial reduction in resistance across all CNT-reinforced specimens following the alignment process. Initially, measured resistance values were recorded for each specimen, and after applying the alignment procedure, a significant decrease was observed, particularly in lower CNT contents.

In particular, for the CNT content of 0.25 wt%, the resistivity decreased by a factor of 4.83, shifting from an average of 14,000 Ω to 2895 Ω after alignment. In comparison, at 1 wt%, the resistivity decreased by a factor of 4.21, from 652 Ω to 154.7 Ω after alignment.

These results indicate that the alignment procedure effectively reduced electrical resistance at all contents of CNTs. However, higher loadings typically showed slightly lower initial and post-alignment resistance values. The most substantial percentage reductions were observed in samples with 0.25 wt% and 0.5 wt% CNT contents. Although the resistance reduction factor after alignment appeared similar for 0.25 wt% and 1 wt% CNT contents, the underlying mechanisms differ. At low CNT content, alignment primarily enabled the initial formation of percolation paths, resulting in a sharp relative resistance drop. In contrast, at 1 wt%, the network was already partially conductive before alignment, and while alignment improved directional conductivity, further gains were limited by CNT agglomeration and the saturation effect. These findings suggest that CNT alignment is particularly effective in samples with less interconnected CNT networks prior to alignment.

The data in Table 1 shows that the alignment process led to increased conductivity across all contents of CNTs. For instance, at 0.25 wt% CNT content, conductivity increased from 1.77 × 10^−5^ S/mm to 8.64 × 10^−4^ S/mm, while for 1 wt%, conductivity increased from 3.83 × 10^−3^ S/mm to 1.62 × 10^−2^ S/mm.

**Figure 5:**
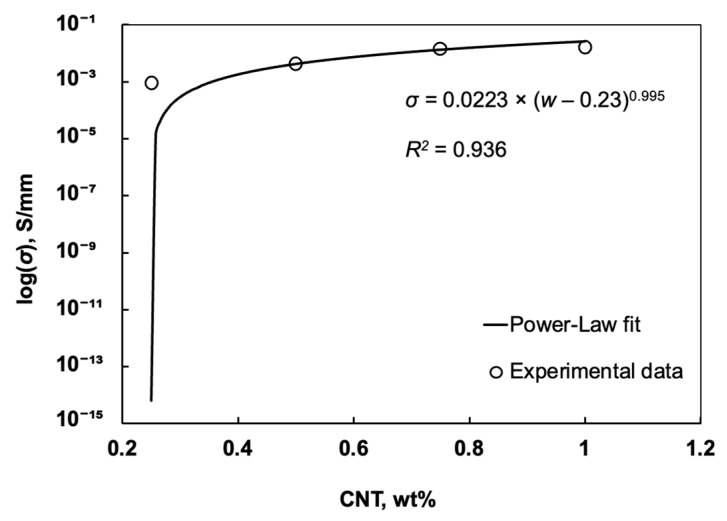
Conductivity vs. CNT weight fraction (after alignment).

Figure 5 illustrates the relationship between CNT weight fraction and electrical conductivity in the epoxy composite after CNT alignment was performed. The data points represent measured conductivity values for each CNT content, while the curve shows the fitted power-law model, which describes the behaviour of electrical conductivity as the CNT content approaches the percolation threshold. The conductivity σ of CNT-enhanced composites follows the percolation model, as expressed in the following equation [36,37]:

*σ* = *A*(*w* − *w_c_*)^*t*^ (2)

where *A* is a constant, *w* is the CNT weight fraction, *w_c_* is the percolation threshold (critical content at which conductivity sharply increases), and *t* is the exponent that describes the conductivity behaviour near the percolation threshold.

Continuous conductive pathway formation occurs within the bonding area when the percolation threshold reaches an estimated 0.23 wt%. Below this threshold, the conductivity remains low because the CNTs are not sufficiently interconnected to allow for an effective electron pathway. The transition from an insulating to a conductive state occurs when the CNT content surpasses 0.23 wt%. This transition becomes evident through the rapid increase in composite conductivity.

The experimental data show that CNT orientation within epoxy results in a lowered percolation threshold, as well as improved conduction properties at lesser additive loadings. This behaviour is beneficial for applications where achieving high conductivity with minimal CNT addition is desired, as it reduces both material costs and potential mechanical compromises due to high filler content.

Wang et al. (2008) [38] reported similar findings, which showed that aligning single-walled carbon nanotubes (SWNTs) in epoxy composites significantly enhanced electrical conductivity compared to non-aligned samples. The electrical conductivity rose from 2 × 10^−10^ S/cm at 0.2 wt% to 6 × 10^−8^ S/cm at 1 wt% because CNT alignment enabled continuous conductive channel formation.

### 3.2. Thermal Analysis of Bonding Area

**Figure 6:**
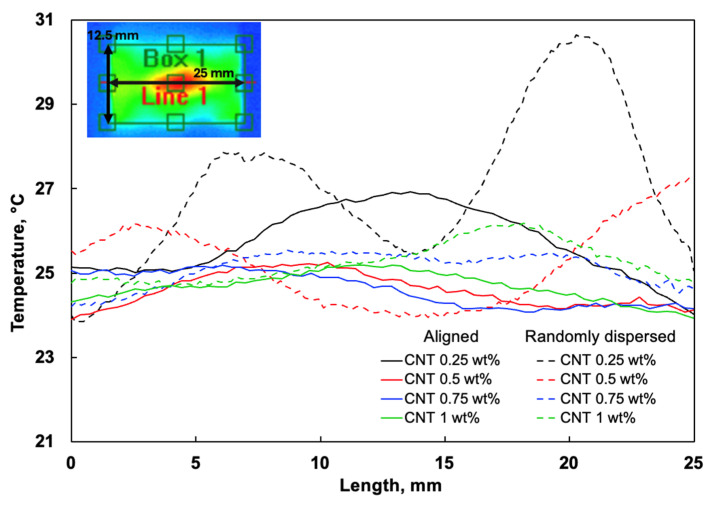
Temperature profile along the length of the aligned and randomly dispersed CNT specimens.

The thermal imaging results depict the temperature distribution across the bonding area at different aligned CNT contents during the curing process. A distinct correlation between CNT loading and heat dissipation efficiency is observed, where increasing CNT content leads to more uniform temperature distribution, reducing localised overheating and enhancing thermal conductivity (Figure 6). This effect is particularly observed at higher CNT concentrations, as improved thermal transport properties facilitate more efficient energy dissipation throughout the adhesive layer. The temperature increase follows a nonlinear trend, with lower CNT contents exhibiting higher temperature differentials, while at higher CNT concentrations, the increase is more moderate. Specifically, at 0.25 wt% CNT, the temperature increases by 2.02 °C (Box 1 area), while at 0.5 wt%, the increase is 1.87 °C. At 0.75 wt%, the observed increase is 1.82 °C, and at 1 wt% CNT, the temperature increase is only 1.41 °C. The diminishing temperature differential at higher CNT loadings suggests the presence of thermal saturation effects, likely caused by CNT agglomeration disrupting the conductive network and altering heat flux uniformity.

To further analyse the temperature distribution during experimental thermal analysis, a line measurement was taken along the length of each specimen, passing through the hottest point, as shown in Figure 6. This temperature profile graph highlights the temperature gradient across the bonding area for each CNT content. The profiles confirm that the specimens with higher CNT contents (especially 1 wt%) exhibited a more uniform temperature distribution along their length, while those with lower CNT contents showed greater temperature variation.

In contrast, specimens with randomly dispersed CNTs showed more pronounced overheating, particularly at low CNT contents (0.25–0.5 wt%). This behaviour stems from the lack of continuous conductive pathways, which restricts Joule heating efficiency and leads to inconsistent curing conditions compared to aligned CNT samples. For improved visualisation, thermal images of randomly dispersed CNT specimens are included in Figure S3.

The thermal analysis of the bonding area indicates that the inclusion of CNTs enhances both thermal conductivity and thermal stability, particularly up to 0.5 wt%. Beyond this content, the thermal benefits diminish as agglomeration effects create voids and irregularities, which reduce bonding uniformity and structural integrity.

To further evaluate the temperature distribution, the numerical modelling results (discussed in Section 3.5.3) were compared to the experimental thermal imaging data.

### 3.3. Single-Lap Shear Strength Tests

**Figure 7:**
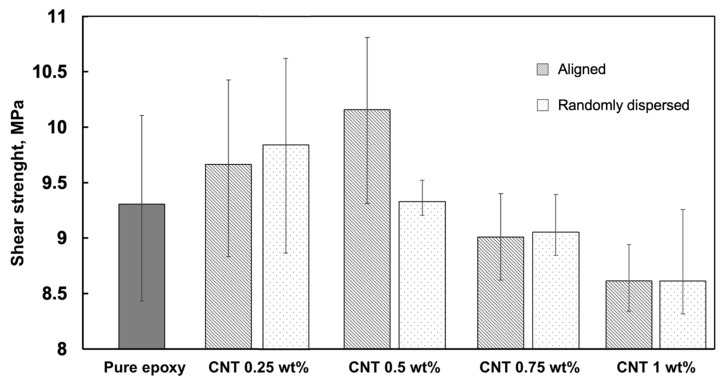
Comparison of shear strength of single-lap specimens for aligned and randomly dispersed CNT loadings and pure epoxy adhesives.

To assess the bonding performance, tensile lap shear strength tests were performed on aligned CNT specimens. Figure 7 illustrates the average shear strength sustained by each type of configuration. Among the CNT-reinforced single-lap samples, the 0.25 wt% and 0.5 wt% CNT contents exhibited the highest shear strength, reaching 9.6 MPa and 10.16 MPa, respectively. Notably, the 0.5 wt% CNT demonstrated the highest strength among the CNT-reinforced samples, indicating that it provides an optimal balance between mechanical integrity and electrical conductivity.

As CNT content increased beyond 0.5 wt%, a slight reduction in shear strength was observed, with values decreasing to 9.01 MPa for 0.75 wt% CNT and 8.6 MPa for 1 wt% CNT. Meanwhile, pure epoxy samples, included as a baseline, exhibited an average shear strength of 9.31 MPa, highlighting the initial improvement achieved with CNT reinforcement at lower contents. However, at higher CNT concentrations, a declining trend in shear strength suggests potential matrix disruptions, likely due to CNT agglomeration effects, increased void percentage, and stress concentration points between the neighbouring CNT particles.

Oven-cured specimens with randomly dispersed CNTs showed similar strength values, with the highest average strength of 9.84 MPa at 0.25 wt% CNT and the lowest of 8.61 MPa at 1 wt% CNT. These results confirm sufficient curing and reinforce the trend observed with aligned CNT samples, where excessive CNT loading may negatively affect mechanical performance.

### 3.4. Post-Fracture SEM Analysis

**Figure 8:**
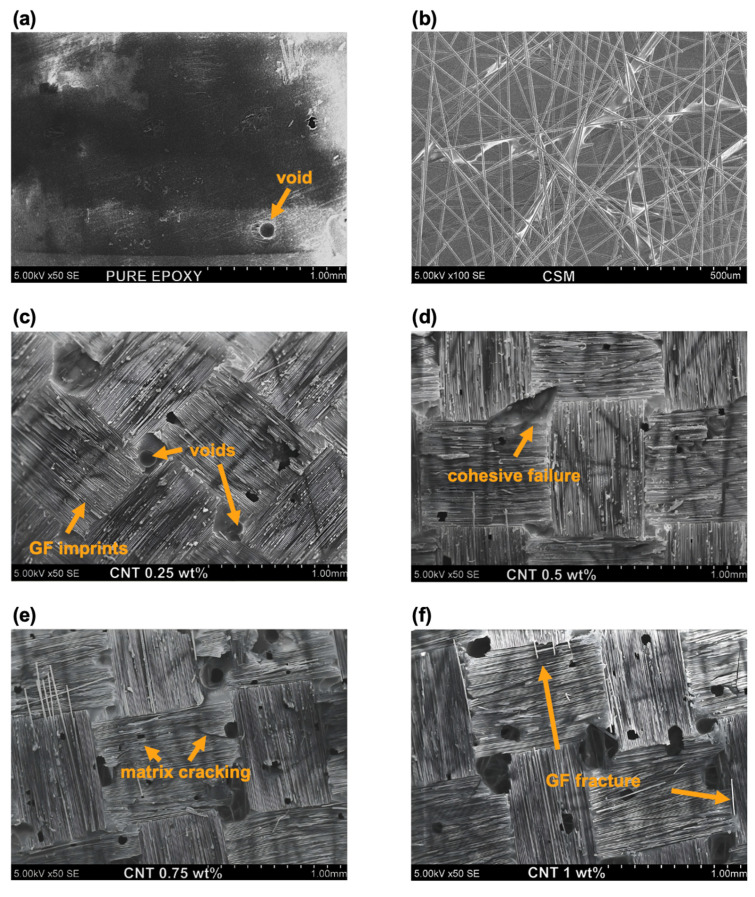
SEM results after lap shear tests: (**a**) pure epoxy adhesive, (**b**) carbon fibre CSM, (**c**) CNT 0.25 wt%, (**d**) CNT 0.5 wt%, (**e**) CNT 0.75 wt%, (**f**) CNT 1 wt%.

SEM was used to analyse the bonding areas of aligned CNT composite samples after shear testing. The SEM images revealed details about the fracture surfaces, adhesion behaviour, and void formation across different CNT contents (Figure 8). The failure modes observed across the specimens primarily indicated cohesive failure, characterised by fracture propagation within the adhesive layer rather than at the adhesive–substrate interface. However, as the CNT content increased, additional microstructural features such as void formation and agglomeration effects became more frequent, influencing the mechanical integrity of the bond.

The fracture surface of pure epoxy (Figure 8a) exhibits a relatively smooth and featureless morphology, indicative of adhesive failure at the specimen substrate level. A carbon fibre CSM, used as a conductive top and bottom layer for CNT-enhanced epoxy specimens, is presented in Figure 8b.

At 0.25 wt% and 0.5 wt% CNT (Figure 8c,d), the fracture surfaces exhibit well-defined glass fibre imprints, suggesting strong adhesion between the adhesive and the composite substrate. The fracture morphology is indicative of cohesive failure, with relatively smooth fracture planes and small voids, confirming effective load transfer and uniform stress distribution. As CNT content increases beyond 0.5 wt% (Figure 8e,f), notable changes in the fracture morphology emerge. At 0.75 wt% and 1 wt% CNT, the SEM images reveal irregular fracture surfaces, an increase in void size, and signs of localised detachment, indicating a loss of bond uniformity. This suggests that excessive CNT content may lead to agglomeration, disrupting the adhesive matrix continuity and creating stress concentration points. These effects are further corroborated by the presence of larger voids and extensive matrix cracking, which could have contributed to the reduction in shear strength observed in mechanical testing.

The observed voids in the fracture surfaces likely originate from multiple factors. One possible explanation is the entrapment of gas or volatile compounds during adhesive curing with higher CNT loading, which affects viscosity and prevents proper resin impregnation. Additionally, CNT agglomeration may introduce regions of poor CNT dispersion, leading to heterogeneous stress distribution and micro-void nucleation under mechanical loading. These voids act as crack initiation sites, reducing overall bond strength and promoting premature failure [39,40]. Additionally, the void surface and amount in the epoxy adhesive calculations show that, depending on the CNT loading, void amount ranges from 100 to 300 (in 36 mm^2^ testing area). The pure epoxy has 1.54% void coverage, while 0.25–1 wt% CNT samples show 2.33%, 6.77%, 9.92%, and 13.10%, respectively (Table 2). The results support the claim that void content increases with higher CNT loadings. Representative images are provided in the Supplementary Materials (Figure S4) for reference. Considering that the mechanical properties are similar between the specimens with aligned and randomly dispersed CNTs, it may be assumed that CNT orientation has minimal influence on void formation.

The transition from predominantly cohesive failure at lower CNT contents (0.25–0.5 wt%) to more irregular and void-rich fracture surfaces at higher CNT loadings (0.75–1 wt%) supports the conclusion that an optimal CNT content exists for balancing mechanical reinforcement and adhesive performance. While lower CNT loadings contribute to enhanced mechanical properties and uniform stress transfer, excessive CNT loading disrupts the adhesive microstructure, resulting in localised defects that negatively impact bonding performance.

**Figure 9:**
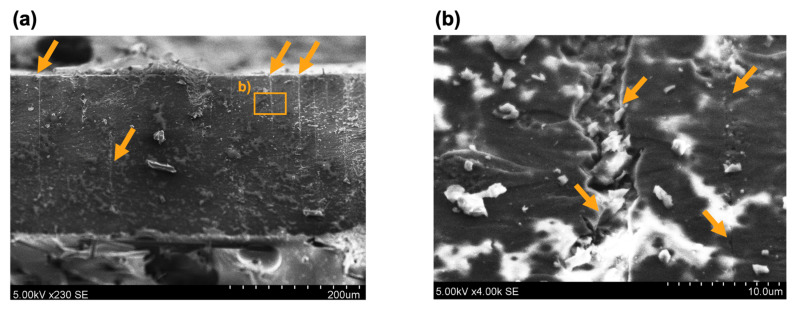
SEM results after lap shear tests of fractured epoxy adhesive with aligned 0.25 wt% CNTs: (**a**) cross-section view, (**b**) magnified area of cracked CNT cluster. Orange arrows indicate areas of CNT clustering (**a**) and fracture propagation paths (**b**).

Additionally, SEM was used to observe the CNT cluster formation of 0.25 wt% fractured lap shear sample. Figure 9a shows a cross-sectional view through the fractured thickness (~220 µm), highlighting the presence of vertical CNT clusters at the cracked surface. On average, it results in one cluster per 60 µm (Figure S5). The fracture surface exhibits localised regions of CNT accumulation, with cracks preferentially propagating along these CNT-rich domains. This suggests that CNT alignment within the matrix modifies the stress distribution, potentially influencing the adhesive bond’s mechanical performance and failure mechanisms.

Figure 9b presents a fractured aligned CNT cluster, where cracks interact with the CNT network. The thickness variations in CNT clustering suggest that fracture behaviour is not entirely uniform across the adhesive layer. The crack surface of the epoxy with more dispersed CNT regions exhibits a smoother fracture, whereas vertical CNT clusters tend to develop void-rich failure zones, leading to stress concentration points. This explains the observed reduction in mechanical performance at higher CNT loadings, where excessive clustering creates discontinuous fracture planes, reducing overall adhesion strength. These findings align with previous observations in Figure 9, indicating that CNT distribution plays a crucial role in governing fracture morphology and mechanical integrity.

### 3.5. Transient Thermal FEA

To establish a clear understanding of the heat transfer mechanisms in CNT-enhanced adhesives, RVE modelling was performed and analysed to better evaluate the processes occurring during the experiment and the obtained results, providing insights into localised Joule heating and thermal conductivity distribution.

#### 3.5.1. Influence of CNT Alignment on Thermal Gradients

**Figure 10:**
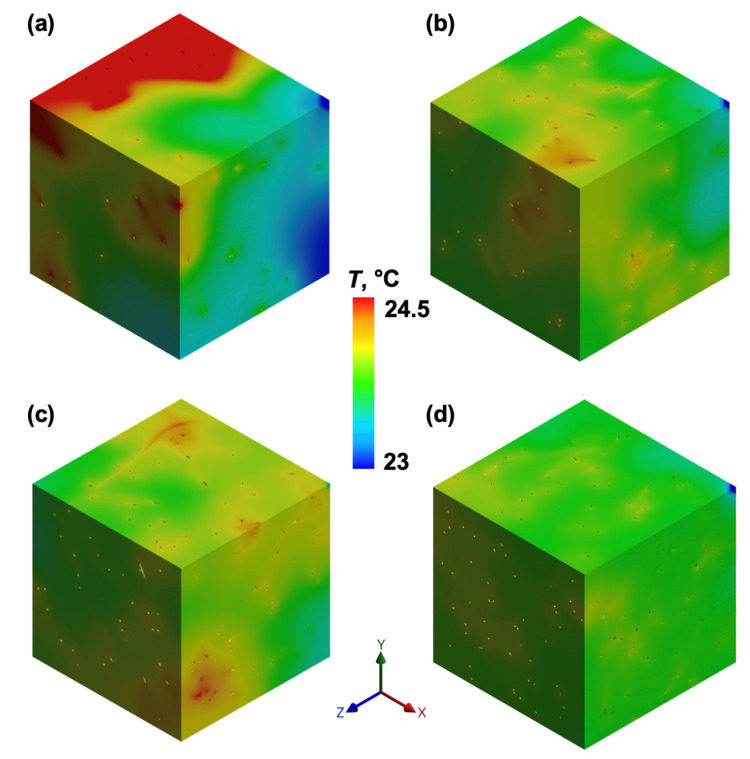
FEA temperature distribution in randomly dispersed CNT loadings: (**a**) 0.25 wt%, (**b**) 0.5 wt%, (**c**) 0.75 wt% (Video S1), and (**d**) 1 wt%.

The formation of thermal gradients was strongly influenced by CNT arrangement. In randomly dispersed CNT models (Figure 10), heat dissipation occurred broadly across the adhesive matrix, leading to a more uniform but less concentrated thermal distribution, as seen in the gradual temperature transitions throughout the RVE domains. At lower CNT loadings, especially 0.25 wt% (Figure 10a), higher temperature variations were observed. At the 0.5 wt% and 0.75 wt% CNT loadings, thermal conductivity was slightly improved, but the heat flux remained relatively dispersed, with no significant localisation effects (Figure 10b,c). At the highest CNT loading (1 wt%), the overall temperature distribution became similar to the average temperature of the model.

**Figure 11:**
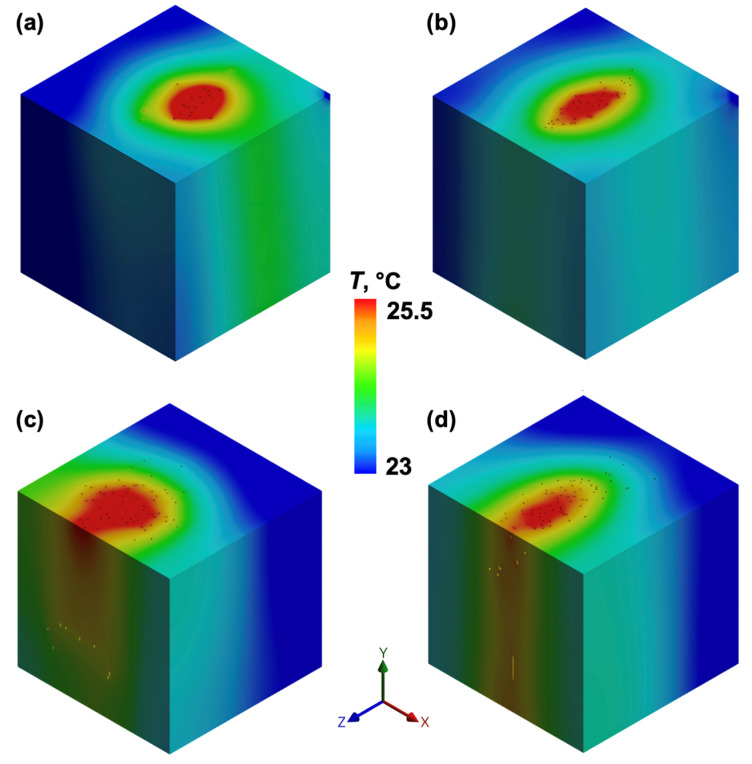
FEA temperature distribution in aligned CNT loadings: (**a**) 0.25 wt%, (**b**) 0.5 wt% (Video S2), (**c**) 0.75 wt%, and (**d**) 1 wt%.

In contrast, Figure 11 illustrates the aligned CNT configurations, where heat is concentrated within well-defined pathways. The structured CNT alignment enhances heat localisation within conductive pathways, leading to sharper thermal gradients compared to the random network. The heat accumulation effect is more evident at lower CNT contents (0.25 wt% and 0.5 wt%), while at higher CNT loadings, the temperature differences become less distinct.

Beyond the alignment effect, cluster position factors also influence the observed temperature distributions in aligned CNT networks. Variations in heating surface exposure and proximity to the outer boundaries affect heat dissipation, contributing to localised temperature differences. A larger effective heating area allows more uniform energy transfer, yet it also results in higher peak temperatures in some cases, e.g., edge proximity effect (Figure 11a—cluster is in the centre of RVE, Figure 11c,d—clusters are closer to the outer boundary of the model). Additionally, when the heating region is closer to the outer edge of the volume, heat dissipation is reduced, further amplifying local temperature variations compared to the randomly distributed CNT configurations.

This trend suggests that excessive CNT concentrations may introduce agglomeration effects, diminishing the efficiency of the conduction network, while the spatial positioning of the heat source plays an equally important role in temperature distribution.

#### 3.5.2. Temperature Distribution and Heat Transfer

**Figure 12:**
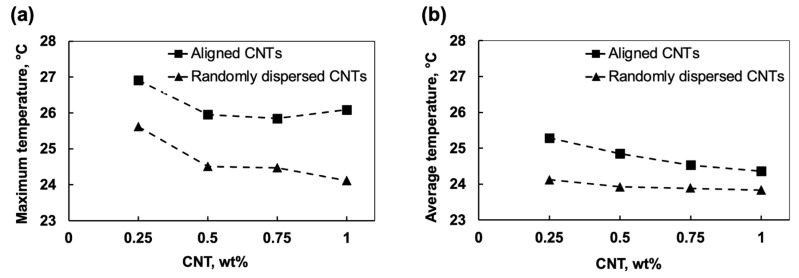
Transient thermal FEA results of CNT modified RVE models: (**a**) maximum and (**b**) average temperatures for different CNT distributions and wt% loading.

Temperature distribution across the adhesive matrix varied significantly based on CNT loading and alignment (Figure 12). The results of maximum and average temperatures indicate that the aligned CNT models facilitated higher localised temperatures, while random distributions resulted in a broader, more uniform spread of heat. The highest peak temperatures were observed in aligned models, reaching 26.91 °C in the 0.25 wt% aligned configuration, compared to 25.61 °C in its random counterpart (Figure 12a). In comparison, at 1 wt% CNT, the peak temperature in the aligned models was 26.09 °C, while in randomly dispersed CNTs, it was 24.11 °C. These results indicate that while alignment enhanced localised heat transfer, higher CNT concentrations did not necessarily lead to a continuous increase in peak temperatures due to potential agglomeration effects.

As shown in Figure 12b, the average temperature followed the same pattern, where the 0.25 wt% aligned model had the highest value of 25.29 °C, compared to 24.12 °C in the random configuration. Meanwhile, for the 1 wt% CNT model, the aligned and random distributions exhibited average temperatures of 24.36 °C and 23.83 °C, respectively.

These differences confirm that alignment leads to localised heat accumulation, enhancing heat transfer through directed conduction pathways, while random dispersions result in a more uniform but less efficient heat distribution. Notably, beyond 0.5 wt% CNT loading, the temperature differences between aligned and random models become less noticeable, suggesting that excessive CNT loading may introduce thermal saturation or clustering effects that diminish further conductivity improvements.

#### 3.5.3. Correlation with Experimental Thermal Imaging

**Figure 13:**
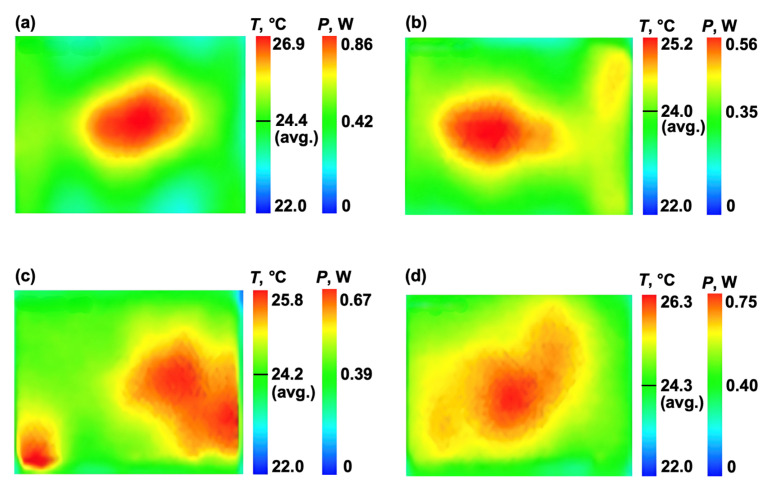
Temperature and power distribution during curing for aligned CNT specimens at: (**a**) 0.25 wt%, (**b**) 0.5 wt%, (**c**) 0.75 wt%, (**d**) 1 wt% loading (heating area 25 × 12.5 mm).

To further investigate the underlying heat distribution mechanisms, experimental thermal imaging results were compared with FEA simulations, where modelled power dissipation values were mapped onto the experimental thermal data (Figure 13). Since Joule heating efficiency is directly related to electrical conductivity, the power dissipation per unit area follows the current–resistance relationship, where localised resistance is strongly influenced by CNT percolation.

The comparison between experimental infrared thermal imaging and simulated heat flux distributions reveals a clear dependence on CNT network structure. Regions exhibiting lower temperatures in the experimental thermal maps correspond to areas where the CNT alignment is not fully developed. This leads to reduced electrical conductivity and lower current density, which diminishes localised heat generation. This behaviour is consistent with FEA simulations of random CNT distributions, where electrical resistivity is higher, and heat dissipation occurs more uniformly rather than being concentrated in specific pathways.

Conversely, the experimental data confirm that areas exhibiting the highest temperatures correspond to regions where CNT alignment facilitated improved electrical conductivity, resulting in increased Joule heating intensity. This correlation aligns with the FEA results, where structured CNT networks exhibited higher localised heat flux, leading to sharper thermal gradients. Notably, variations in heating surface exposure and proximity to the outer boundaries influence heat dissipation, contributing to localised temperature differences. The effective heating surface area is larger in some cases, allowing for more uniform energy transfer and higher peak temperatures.

The decreasing temperature differentials observed at higher CNT loadings suggest a complex interaction between network formation and thermal transport mechanisms. The reduction in temperature variation at 1 wt% CNT implies a possible saturation point, beyond which additional CNT loading does not linearly enhance thermal performance. This behaviour is likely associated with CNT clustering, which may disrupt percolation pathways and increase localised resistive heating. Theoretical predictions suggest that an optimal CNT concentration exists, balancing electrical conductivity improvements with thermal dissipation efficiency [41]. At lower concentrations, the primary limitation is insufficient CNT percolation, leading to higher resistive losses and dispersed heat dissipation. As CNT content increases, the formation of conductive pathways enhances localised heat transfer. However, excessive CNT loading beyond the percolation threshold can introduce agglomeration effects, leading to the formation of resistive regions that reduce the overall efficiency of the heating network.

It is important to note that the finite element model was designed to represent only the CNT-enhanced adhesive layer using a simplified representative volume element (RVE) and does not incorporate additional materials present in the experimental setup, such as the carbon CSM or silver conductive ink. These external layers primarily affect current injection and boundary conductivity, whereas the simulated power distribution focuses on the internal Joule heating behaviour within the CNT-epoxy matrix. The comparative analysis in Figure 13 aims to highlight the relative differences in localised heating effects created by CNT distribution, rather than replicate the full multi-material stack. This modelling approach allowed us to isolate the contribution of CNT alignment to thermal behaviour with reduced computational complexity, while still capturing key experimental trends.

## 4. Conclusions

This study explored the use of CNT-enhanced adhesives for localised Joule heating-based curing in composite bonding. CNT-reinforced epoxy adhesives (0.25–1 wt%) were evaluated for their electrical, thermal, and mechanical performance. Electrical resistance measurements demonstrated a significant reduction after alignment. Conductivity increased from 1.77 × 10^−5^ S/mm for a random 0.25 wt% CNT sample to 8.64 × 10^−4^ S/mm for aligned. The percolation threshold was determined at 0.23 wt%, beyond which electrical conductivity increased sharply. Lap shear tests identified 0.5 wt% CNT as optimal, achieving a shear strength of 10.16 MPa, an increase compared to pure epoxy resin (9.31 MPa). In contrast, higher loadings (1 wt%) led to agglomeration and a reduction in strength to 8.6 MPa, falling slightly below the baseline in both the aligned and randomly dispersed CNT specimens. SEM analysis revealed a formation of vertical CNT clusters and a shift from cohesive failure at lower CNT loadings to void-rich fracture patterns at higher concentrations. FEA and infrared thermal imaging confirmed that CNT alignment enhanced heat localisation, leading to concentrated heating zones instead of uniform heat distribution. While alignment improved targeted heating efficiency, excessive CNT content introduced agglomeration, reducing heat transfer uniformity. FEA simulations indicated that aligned CNTs enhanced heat transfer, with localised temperature peaks of 26.91 °C at 0.25 wt%, while random CNT dispersion resulted in lower peak temperatures of 25.61 °C and broader heat distribution. Additionally, the work presented power distribution mapping at the adhesive layer using the inverse modelling technique. These results show that CNT-enhanced Joule heating enables targeted, energy-efficient curing with improved mechanical properties of a single-lap joint.

## Figures and Tables

**Table 1:** Calculated conductivity values before and after alignment.

CNT	0.25 wt%	0.5 wt%	0.75 wt%	1 wt%
Conductivity before alignment, S/mm	1.77 × 10^−5^	2.15 × 10^−4^	2.29 × 10^−3^	3.83 × 10^−3^
Conductivity after alignment, S/mm	8.64 × 10^−4^	4.20 × 10^−3^	1.41 × 10^−2^	1.62 × 10^−2^

**Table 2:** Quantitative surface analysis of void content at different CNT loadings.

Specimen Type	Pure Epoxy	CNT 0.25 wt%	CNT 0.5 wt%	CNT 0.75 wt%	CNT 1 wt%
Void area, %	1.54	2.33	6.77	9.92	13.10

## Data Availability

Data are contained within the article and Supplementary Materials.

## Supplementary Materials

The following supporting information can be downloaded at: https://www.mdpi.com/article/10.3390/polym17091176/s1, Figure S1: Infrared thermography setup for experimental testing; Figure S2: Single-lap testing setup used for mechanical evaluation; Figure S3: Thermal imaging results of randomly dispersed specimens with CNT content: (a) 0.25 wt%, (b) 0.5 wt%, (c) 0.75 wt%, (d) 1 wt%; Figure S4: Void formation in specimens with CNT content: (a) 0.25 wt%, (b) 0.5 wt%, (c) 0.75 wt%, (d) 1 wt% and (e) pure epoxy (image area 3 × 4 mm, total tested area: 36 mm2); Figure S5: SEM images of 0.25 wt% CNT loading specimen cluster quantification; Video S1: Video of temperature distribution in RVE with randomly dispersed CNTs; Video S2: Video of temperature distribution in RVE with aligned CNTs.

## Abbreviations

The following abbreviations are used in this manuscript:

CNT	Carbon Nanotube
CSM	Chopped Strand Mat
DC	Direct Current
FEA	Finite Element Analysis
GF	Glass Fibre
GFRP	Glass Fibre-Reinforced Polymer
ISO	International Organization for Standardization
PWM	Pulse Width Modulation
RVE	Representative Volume Element
SEM	Scanning Electron Microscopy
