# Supplementary material for: Electrically Conductive Nanoparticle-Enhanced Epoxy Adhesives for Localised Joule Heating-Based Curing in Composite Bonding
Source: Polymers (Basel). 2025 Apr 25;17(9):1176. doi: 10.3390/polym17091176 (PMC12073144; doi:10.3390/polym17091176)
Supplement: Supplementary file 1 [file polymers-17-01176-s001.zip › Supplementary Materials.pdf]

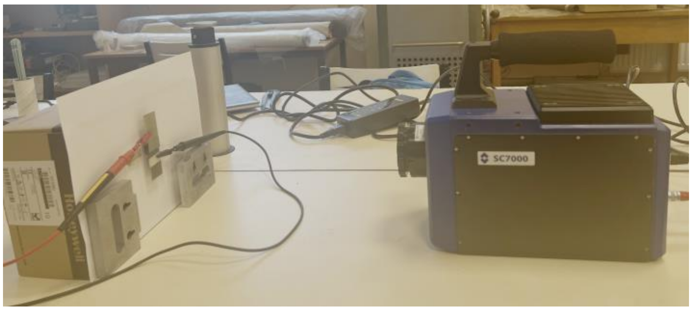

Figure S1. Infrared thermography setup for experimental testing

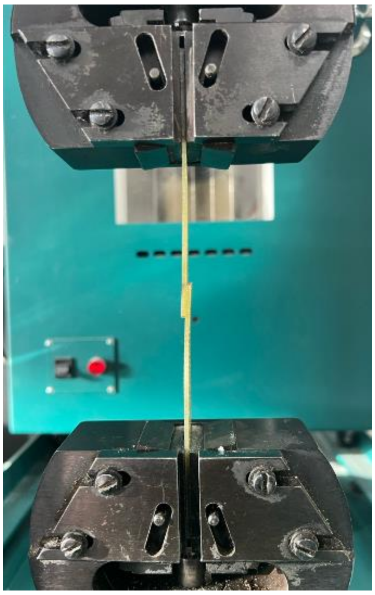

Figure S2. Single-lap testing setup used for mechanical evaluation

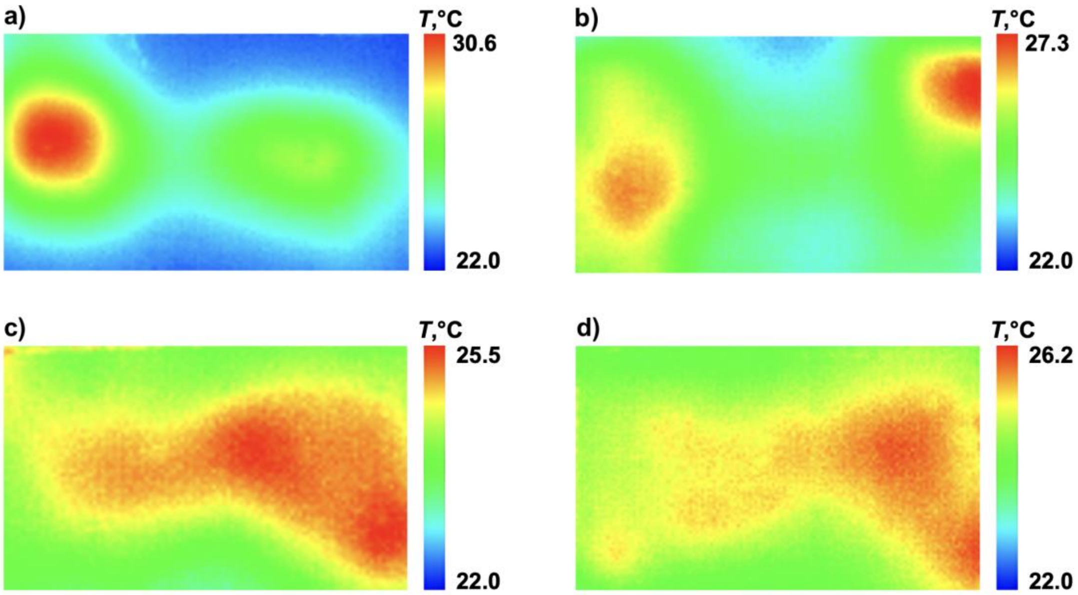

Figure S3. Thermal imaging results of randomly dispersed specimens with CNT content: a) 0.25 wt%, b) 0.5 wt%, c) 0.75 wt%, d) 1 wt%

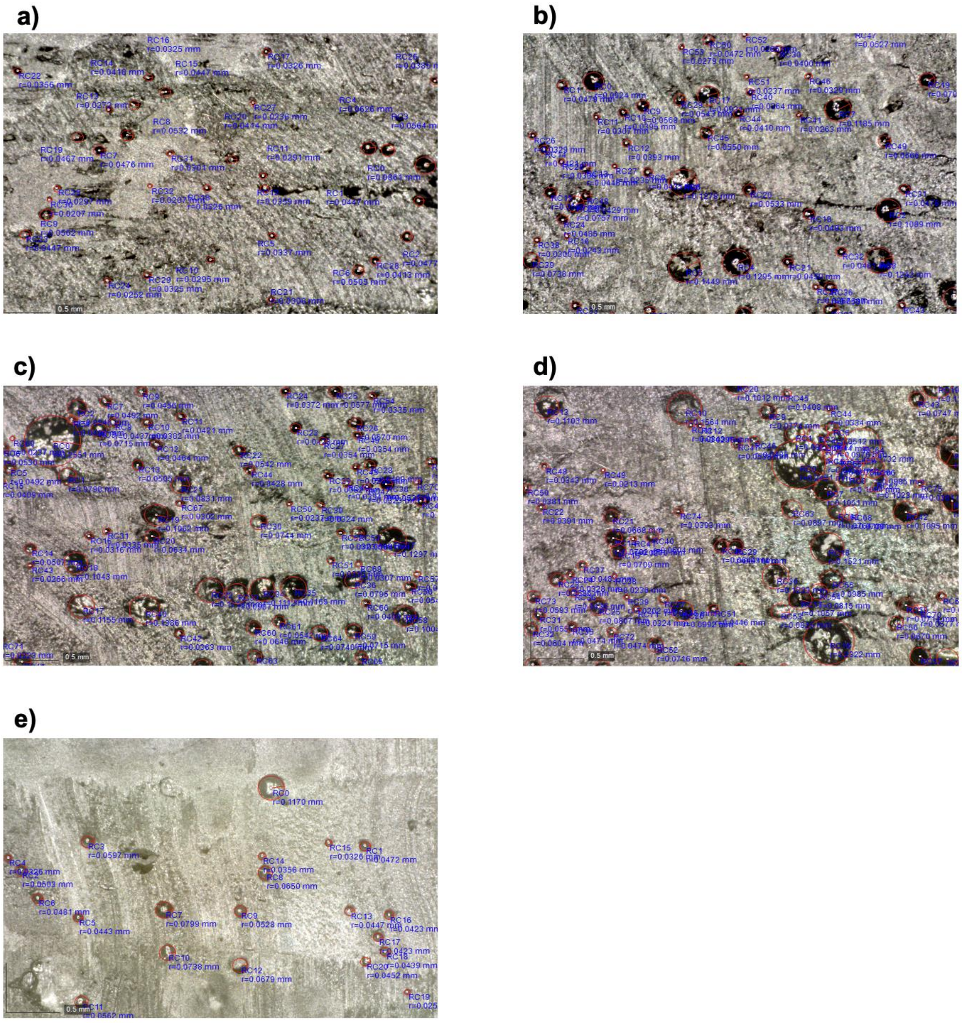

Figure S4. Void formation in specimens with CNT content: a) 0.25 wt%, b) 0.5 wt%, c) 0.75 wt%, d) 1 wt% and e) pure epoxy (image area 3 × 4 mm, total tested area: 36 mm^2)

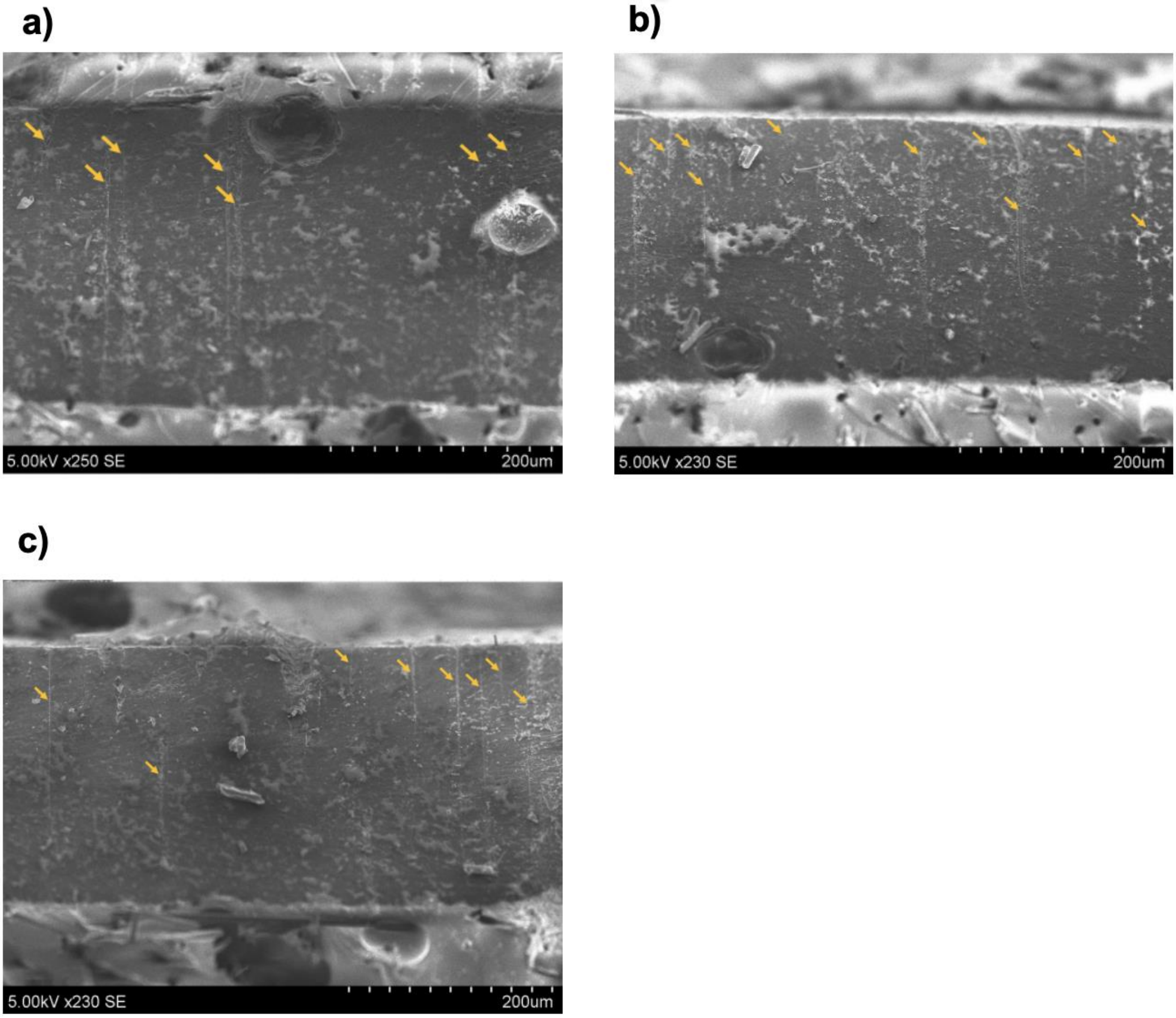

Figure S5. SEM images of 0.25 wt% CNT loading specimen cluster quantification
